# Associations of dual use and conventional cigarette smoking with metabolic syndrome in Korean adults: a population-based study

**DOI:** 10.3389/fpubh.2026.1833270

**Published:** 2026-06-03

**Authors:** Min-Hee Park, Bomi An

**Affiliations:** 1 College of Nursing, Wonkwang University, Iksan-si, Jeonbuk, Republic of Korea; 2 Department of Nursing, Hannam University, Daejeon, Republic of Korea

**Keywords:** dual use, electronic cigarettes, metabolic syndrome, smoking, smoking cessation

## Abstract

**Introduction:**

Despite the recent increase in the number of e-cigarette users, the association between smoking patterns involving e-cigarette use, particularly dual use with conventional cigarettes, and metabolic syndrome (MetS) remains poorly explored. This study examined the association of dual use and cigarette-only smoking with MetS relative to non-smoking.

**Methods:**

This study involved 4,942 adults aged ≥19 years from the Korea National Health and Nutrition Examination Survey (2023). Participants were classified into non-smokers (*n* = 4,156, 81.2%), cigarette-only smokers (*n* = 625, 14.3%), and dual users (*n* = 161, 4.4%), defined as current smokers of conventional cigarettes who concurrently use either heated tobacco products (HTPs) or liquid e-cigarettes. Multivariate logistic regression analysis was used to assess associations between smoking types and MetS.

**Results:**

The odds of MetS were significantly higher in dual users (OR = 1.50, 95% CI = 1.02–2.19, *p* = 0.038) and cigarette-only smokers (OR = 1.41, 95% CI = 1.10–1.81, *p* = 0.008) than in non-smokers. Considering MetS components, the odds of hypertriglyceridemia was higher in dual users (OR = 2.16, 95%CI = 1.46–3.18, *p* < 0.001) and cigarette-only smokers (OR = 1.91, 95% CI = 1.54–2.36, *p* < 0.001) than in non-smokers; the odds of low high-density lipoprotein cholesterol levels was higher only in cigarette-only smokers (OR = 2.32, 95% CI = 1.78–3.02, *p* < 0.001).

**Conclusion:**

Both dual users and cigarette-only smokers showed higher odds of MetS compared with non-smokers. Hypertriglyceridemia was associated with both smoking groups, whereas low HDL cholesterol was associated only with cigarette-only smokers. Public health strategies should continue to support smoking cessation and improve awareness regarding the potential metabolic risks associated with smoking behaviors.

## 1 Introduction

Metabolic syndrome (MetS) is characterized by the presence of five major risk factors associated with the increased risk of cardiovascular disease: abdominal obesity, hypertension, hyperglycemia, low high-density lipoprotein (HDL) cholesterol levels, and triglyceridemia (1). The proportion of patients with MetS varies slightly depending on the MetS definition applied, which is based on the characteristics of the population in each region, and the reported prevalence of MetS ranges from as low as 12.5% to as high as 31.4% (2). Cardiovascular diseases, including ischemic heart disease and stroke, are well-recognized as the leading cause of death among non-communicable diseases worldwide (3). Therefore, proactive prevention of MetS is crucial to prevent cardiovascular disease. MetS is closely associated with modifiable lifestyles such as drinking, smoking, and exercise. In particular, conventional cigarette smoking has been shown to be associated with abdominal obesity, blood pressure, blood glucose concentrations, and blood lipid profiles and, hence, has been associated with MetS (4–6).

The World Health Organization (WHO) reported that the number of adults consuming tobacco worldwide decreased from 32.7% in 2000 to 22.3% in 2020 and is expected to continue declining (7). The decline is generally attributed to the implementation of tobacco control policies (8). However, despite this progress, the number of e-cigarette users is growing, with an estimated 82 million users globally (9). In particular, considering the usage status of e-cigarette users, a high proportion of e-cigarette users, approximately 90%, are dual users who use both e-cigarettes and conventional cigarettes (10–12). Therefore, it is necessary to investigate the metabolic health associations observed among dual users of conventional cigarettes and e-cigarettes.

To the best of our knowledge, only a few studies have examined the association between MetS and dual use conducted to date (12–17). Previous studies have reported that both dual use and conventional smoking have been associated with an increased prevalence of MetS compared with non-smoking (13–17); however, the results were slightly inconsistent when examined by sex (16, 17). Conversely, one study reported that there was no association between dual use and the risk of MetS (12). Regarding the five components of MetS, previous studies have identified significant associations between dual use and abdominal obesity (12, 17), low HDL cholesterol (13, 17), and hypertriglyceridemia (13, 16). Given the inconsistent results regarding MetS components, further investigation is needed to better understand the associations between dual use, MetS, and its individual components. Accordingly, we aimed to investigate the risk of MetS in dual users and conventional cigarette-only users, each relative to non-smokers. The specific objectives of this study were as follows:

1. To investigate the differences in general and health-related characteristics of non-smokers, cigarette-only smokers, and dual users.
2. To identify the prevalence of MetS and its five components across the three smoking groups (non-smokers, cigarette-only smokers, and dual users).
3. To analyze the association between each smoking type (dual use and cigarette-only use) and the likelihood of MetS and its individual components, using non-smokers as the reference group.

## 2 Methods

### 2.1 Study design

This descriptive study analyzed secondary data from the Korea National Health and Nutrition Examination Survey (KNHANES). The KNHANES is a statutory survey that explores health behavior, the prevalence of chronic diseases, and food and nutrition intake status among Koreans and is conducted under Article 16 of the Korea National Health Promotion Act.

### 2.2 Participants

Of the 6,929 participants in the 2023 KNHANES, 4,942 were included in the final analysis. The selection process followed strict exclusion criteria to ensure the validity of the comparison among non-smokers, cigarette-only smokers, and dual users. First, we excluded individuals under 19 years of age (*n* = 1,897). Second, participants with missing values for key variables, such as smoking status or components of metabolic syndrome (*n* = 90), were removed. Consequently, the final 4,942 participants were classified into three distinct groups: (1) Non-smokers, defined as individuals who either have never smoked or are former smokers who do not currently smoke; (2) Cigarette-only smokers, defined as current smokers of conventional cigarettes who use neither heated tobacco products (HTPs) nor liquid e-cigarettes; and (3) Dual users, defined as current smokers of conventional cigarettes who also use either HTPs or liquid e-cigarettes.

### 2.3 Variables

This study utilized some items regarding general characteristics, health behaviors, and MetS among the KNHANES variables. The details are as follows.

#### 2.3.1 General characteristics

This study included the following general characteristics of participants: sex, age, residential area, marital status, national health insurance, education level, household income, and occupation.

#### 2.3.2 Health-related characteristics

The health-related characteristics included a smoking cessation plan, drinking, walking, resistance exercise, stress, depression, and subjective health status. The smoking cessation plan was categorized as yes or no, depending on whether participants planned to quit smoking within 1 month. Drinking was classified as more than twice a week, 2–4 times a month, once or less a month, and non-drinking; walking as practiced or not practiced, depending on whether participants walked more than five times a week for at least 30 min per session. Resistance exercise was classified as yes or no, depending on whether or not participants performed resistance exercise twice a week. Stress was classified as considerable or mild; depression as yes or no, depending on whether participants experienced depression for more than 2 weeks. Subjective health status was classified as good, moderate, and bad.

#### 2.3.3 MetS

MetS was defined as having three or more of the following components: abdominal obesity, hypertension, hyperglycemia, hypertriglyceridemia, and low HDL cholesterol; non-MetS as fewer than three components. The diagnostic criteria for MetS followed the 2005 Adult Treatment Panel III (ATP III) guidelines of the American Heart Association (AHA)/National Heart Lung and Blood Institute (NHLBI) (18). To reflect the characteristics of the Korean population, abdominal obesity was based on the criteria of the 2018 Korean Society for the Study of Obesity guideline (19). The specific criteria for MetS diagnosis were as follows:

- Abdominal obesity: waist circumference ≥90 cm (male) and ≥85 cm (female)
- Hypertension: systolic blood pressure ≥130 mmHg or diastolic blood pressure ≥85 mmHg
- Hyperglycemia: fasting blood glucose levels ≥100 mg/dL
- Hypertriglyceridemia: triglyceride levels ≥150 mg/dL
- Hypo-HDL cholesterolemia: HDL cholesterol levels <40 mg/dL (male) and <50 mg/dL (female)

### 2.4 Data analysis

Data were analyzed using IBM SPSS Statistics 26.0 (IBM Corp., Armonk, NY, USA). To ensure the representativeness of the South Korean population and account for the complex survey design, we utilized the complex samples analysis module by incorporating strata, primary sampling units (PSUs), and integrated sampling weights as specified in the KNHANES 2023 guidelines. The specific statistical procedures were as follows:

First, the Rao-Scott chi-square test was performed to compare socio-demographic and health-related characteristics among the three groups (non-smokers, cigarette-only smokers, and dual users). This method was specifically employed to account for the complex sampling design and to ensure the validity of *p*-values, particularly for categorical variables with small cell counts in the dual-user group. Second, the prevalence rates of Metabolic Syndrome (MetS) and its five individual components were examined across the three groups using the Rao-Scott chi-square test. Third, multivariable logistic regression analysis was conducted to analyze the association between smoking types and the likelihood of MetS. Smoking cessation plan was not included in the adjusted model because this variable was structurally inapplicable to non-smokers. To ensure model stability and address Events-Per-Variable (EPV) considerations, especially given the sample size of the dual-user group (n = 161), we carefully selected a parsimonious set of covariates based on theoretical relevance and established clinical significance in previous literature (20–22). Model diagnostics were performed to assess the robustness of the logistic regression estimates; the Hosmer-Lemeshow test was used to evaluate the goodness-of-fit, and multicollinearity among covariates was checked using Variance Inflation Factors (VIF). All VIF values were less than 2.0, suggesting no significant issues with multicollinearity. Furthermore, we ensured that the number of events (cases of MetS) was sufficient relative to the number of independent variables to maintain the reliability of the odds ratio estimates. Given the multiple comparisons performed across various MetS components, these analyses were considered exploratory.

### 2.5 Ethical considerations

This study used publicly available data from the Korea National Health and Nutrition Examination Survey (KNHANES). The survey was approved by the Institutional Review Board of the Korea Disease Control and Prevention Agency (KDCA), and the present study was exempt from additional ethical approval because it used de-identified secondary data.

## 3 Results

### 3.1 General characteristics analyzed by smoking type

The general characteristics of participants that differed significantly by smoking type included sex (*χ*^2^ = 615.41, *p* < 0.001), age (*χ*^2^ = 184.42, *p* < 0.001), marital status (*χ*^2^ = 98.08, *p* < 0.001), national health insurance (*χ*^2^ = 51.44, *p* < 0.001), education level (*χ*^2^ = 76.25, *p* < 0.001), household income (*χ*^2^ = 27.52, *p* = 0.013), and occupation (*χ*^2^ = 221.12, *p* < 0.001). Male participants predominated among smokers, with 80.2% of dual users and 86.5% of cigarette-only smokers being men, whereas 60.0% of non-smokers were women. By age group, non-smokers were more common among those aged ≥60 years (33.4%), cigarette-only smokers those aged 50–59 years (26.2%), and dual users were concentrated in younger adults (34.5% at 19–29 years; 26.9% at 30–39 years). Unmarried status was frequent among dual users (52.5%). Regarding health insurance, cigarette-only smokers had a higher proportion of participants covered by medical aid (7.4%). With respect to education, college graduates or higher were most common among non-smokers (46.1%), followed by dual users (45.3%) and cigarette-only smokers (39.6%). Dual users also had the highest proportion in the highest income group (35.6%). In terms of occupation, service/sales (24.2%) and office work (21.3%) were common among dual users, whereas cigarette-only smokers were more often unemployed (24.1%) or technicians (22.3%) (Table 1).

**Table 1:** General characteristics according to smoking type.

Variables	Total	Smoking type			Rao-Scott *χ*^2^	*p*
		Dual user	Cigarette-only smoker	Non-smoker		
Total	4,942 (100.0)	161 (4.4)	625 (14.3)	4,156 (81.2)		
Sex						
Male	2,100 (48.4)	127 (80.2)	529 (86.5)	1,444 (40.0)	615.41	<0.001
Female	2,842 (51.6)	34 (19.8)	96 (13.5)	2,712 (60.0)		
Age (years)						
19–29	513 (15.8)	50 (34.5)	66 (14.9)	397 (15.0)	184.42	<0.001
30–39	562 (15.3)	41 (26.9)	59 (12.2)	462 (15.2)		
40–49	816 (18.0)	44 (24.1)	124 (22.4)	648 (16.9)		
50–59	967 (20.1)	19 (11.3)	147 (26.2)	801 (19.5)		
≥60	2,084 (30.8)	7 (3.1)	229 (24.3)	1,848 (33.4)		
Residence						
Urban	3,934 (83.2)	141 (87.7)	477 (80.2)	3,316 (83.5)	8.19	0.070
Rural	1,008 (16.8)	20 (12.3)	148 (19.8)	840 (16.5)		
Marital status						
Married	4,043 (74.7)	80 (47.5)	477 (71.4)	3,486 (76.7)	98.08	<0.001
Unmarried	899 (25.3)	81 (52.5)	148 (28.6)	670 (23.3)		
Health insurance						
Regional NHI	1,596 (30.4)	50 (31.6)	231 (34.5)	1,315 (29.6)	51.44	<0.001
Workplace NHI	3,156 (66.1)	107 (64.4)	343 (58.1)	2,706 (67.6)		
Medical aid	190 (3.5)	4 (4.0)	51 (7.4)	135 (2.7)		
Education						
≤Middle school	1,295 (19.3)	6 (3.3)	135 (16.7)	1,154 (20.7)	76.25	<0.001
High school	1,681 (35.5)	80 (51.4)	273 (43.7)	1,328 (33.2)		
≥College	1,966 (45.2)	75 (45.3)	217 (39.6)	1,674 (46.1)		
Household income						
Low	918 (14.7)	14 (8.1)	119 (16.0)	785 (14.9)	27.52	0.013
Mid-low	1,198 (22.5)	22 (15.8)	177 (26.2)	999 (22.2)		
Mid-high	1,373 (30.2)	62 (40.5)	165 (26.9)	1,146 (30.2)		
High	1,453 (32.6)	63 (35.6)	164 (31.0)	1,226 (32.7)		
Occupation						
Manager-Experts	735 (17.2)	24 (12.7)	87 (15.2)	624 (17.8)	221.12	<0.001
Office worker	508 (12.0)	32 (21.3)	49 (9.6)	427 (11.9)		
Service-Sales	648 (13.3)	37 (24.2)	75 (11.8)	536 (13.0)		
Agriculture-Fisheries	149 (2.2)	2 (0.5)	24 (2.9)	123 (2.2)		
Technician	489 (11.5)	31 (18.9)	124 (22.3)	334 (9.2)		
Simple labor	531 (9.7)	9 (7.1)	90 (14.1)	432 (9.0)		
Unemployed	1,882 (34.1)	26 (15.3)	176 (24.1)	1,680 (36.9)		

Values are presented as numbers (weighted%).

### 3.2 Health-related characteristics analyzed by smoking type

Analyzing health-related characteristics of participants by smoking type, significant differences were found in smoking cessation plan (*χ*^2^ = 636.82, *p* < 0.001), drinking (*χ*^2^ = 343.89, *p* < 0.001), walking (*χ*^2^ = 41.34, *p* < 0.001), stress (*χ*^2^ = 60.07, *p* < 0.001), and depression (*χ*^2^ = 24.51, *p* < 0.001). The proportion of participants with a smoking cessation plan was higher among cigarette-only smokers (16.2%) than dual users (6.2%). Dual users and cigarette-only smokers showed the highest proportion of drinking more than twice weekly (38.3 and 38.7%), while non-smokers had the highest proportion of drinking once or less a month (33.5%). Walking practice was most common among non-smokers (47.0%), followed by dual users (36.7%) and cigarette-only smokers (35.0%). Perceived stress was highest in dual users (41.5%), followed by cigarette-only smokers (31.9%), and non-smokers (22.8%), and a similar pattern was observed for depression, with prevalence rates of 19.5, 12.7, 9.8%, respectively (Table 2).

**Table 2:** Health-related characteristics according to smoking type.

Variables	Total (*n* = 4,942)	Smoking type			Rao-Scott *χ*^2^	*p*
		Dual user (*n* = 161)	Cigarette-only smoker (*n* = 625)	Non-smoker (*n* = 4,156)		
Smoking cessation plan						
Yes	115 (2.6)	10 (6.2)	105 (16.2)	0 (0.0)	636.82	<0.001
No	4,827 (97.4)	151 (93.8)	520 (83.8)	4,156 (100.0)		
Drinking						
≥2 per week	892 (19.3)	62 (38.3)	249 (38.7)	581 (14.8)	343.89	<0.001
2–4 per month	1,056 (23.1)	48 (29.1)	154 (24.5)	854 (22.5)		
≤1 per month	1,527 (31.9)	42 (27.1)	129 (24.0)	1,356 (33.5)		
None	1,467 (25.7)	9 (5.5)	93 (12.7)	1,365 (29.1)		
Walking						
Yes	2,224 (44.9)	59 (36.7)	217 (35.0)	1,948 (47.0)	41.34	<0.001
No	2,718 (55.1)	102 (63.3)	408 (65.0)	2,208 (53.0)		
Resistance exercise						
Yes	1,237 (26.5)	43 (28.3)	142 (24.1)	1,052 (26.8)	2.67	0.372
No	3,705 (73.5)	118 (71.7)	483 (75.9)	3,104 (73.2)		
Stress						
Considerable	1,163 (25.0)	62 (41.5)	185 (31.9)	916 (22.8)	60.07	<0.001
Mild	3,779 (75.0)	99 (58.5)	440 (68.1)	3,240 (77.2)		
Depression						
Yes	530 (10.6)	28 (19.5)	82 (12.7)	420 (9.8)	24.51	<0.001
No	4,412 (89.4)	133 (80.5)	543 (87.3)	3,736 (90.2)		
Subjective health						
Good	1,617 (35.2)	54 (33.2)	187 (31.2)	1,376 (36.0)	8.90	0.171
Moderate	2,389 (47.2)	76 (46.8)	304 (48.5)	2,009 (47.0)		
Bad	936 (17.6)	31 (20.0)	134 (20.3)	771 (17.0)		

Values are presented as numbers (weighted%).

### 3.3 MetS analyzed by smoking type

MetS prevalence differed significantly across smoking type (*χ*^2^ = 40.85, *p* < 0.001). The prevalence of MetS was 23.3% among dual users, 29.8% in cigarette-only smokers, and 19.3% among non-smokers. Significant differences were observed in all MetS components except abdominal obesity, including hypertension (*χ*^2^ = 8.68, *p* = 0.026), hyperglycemia (*χ*^2^ = 24.72, *p* < 0.001), hypertriglyceridemia (*χ*^2^ = 175.44, *p* < 0.001), and low HDL cholesterol (*χ*^2^ = 32.22, *p* < 0.001). The prevalence of hypertension was highest among cigarette-only smokers (24.6%), followed closely by non-smokers (23.4%). Hyperglycemia (40.7%) and low HDL cholesterol (28.2%) were most prevalent among cigarette-only smokers, whereas hypertriglyceridemia showed high prevalent in both dual users (41.6%) and cigarette-only smokers (42.1%) (Table 3).

**Table 3:** Metabolic syndrome according to smoking type.

Variables	Total (*n* = 4,942)	Smoking type			Rao-Scott *χ*^2^	*p*
		Dual user (*n* = 161)	Cigarette-only smoker (*n* = 625)	Non-smoker (*n* = 4,156)		
Metabolic syndrome						
Yes	1,108 (21.0)	39 (23.3)	194 (29.8)	875 (19.3)	40.85	<0.001
No	3,834 (79.0)	122 (76.7)	431 (70.2)	3,281 (80.7)		
Abdominal obesity						
Yes	1,822 (35.4)	62 (36.4)	246 (38.2)	1,514 (34.8)	3.14	0.399
No	3,120 (64.6)	99 (63.6)	379 (61.8)	2,642 (65.2)		
Hypertension						
Yes	1,286 (23.2)	28 (15.2)	175 (24.6)	1,083 (23.4)	8.68	0.026
No	3,656 (76.8)	133 (84.8)	450 (75.4)	3,073 (76.6)		
High glucose						
Yes	1,787 (32.8)	43 (27.6)	278 (40.7)	1,466 (31.7)	24.72	<0.001
No	3,155 (67.2)	118 (72.4)	347 (59.3)	2,690 (68.3)		
Hypertriglyceridemia						
Yes	1,174 (25.0)	65 (41.6)	257 (42.1)	852 (21.1)	175.44	<0.001
No	3,768 (75.0)	96 (58.4)	368 (57.9)	3,304 (78.9)		
Low HDL cholesterol						
Yes	1,034 (20.2)	31 (18.4)	166 (28.2)	837 (18.9)	32.22	<0.001
No	3,908 (79.8)	130 (81.6)	459 (71.8)	3,319 (81.1)		

Values are presented as numbers (weighted%).

### 3.4 Association between smoking types and metabolic syndrome

To examine the association between smoking types and metabolic syndrome (MetS), multivariable logistic regression was performed using non-smokers as the reference group. Covariates were selected based on theoretical relevance and established clinical significance. Both dual users (OR = 1.50, 95% CI = 1.02–2.19, *p* = 0.038) and cigarette-only smokers (OR = 1.41, 95% CI = 1.10–1.81, *p* = 0.008) showed significantly higher odds of MetS compared to non-smokers. Given the multiple comparisons, the findings for individual MetS components are considered exploratory. The adjusted odds of hypertriglyceridemia were significantly higher in both dual users (OR = 2.16, 95%CI = 1.46–3.18, *p* < 0.001) and cigarette-only smokers (OR = 1.91, 95% CI = 1.54–2.36, *p* < 0.001) than in non-smokers. Additionally, a significant association with low HDL cholesterol was observed exclusively among cigarette-only smokers (OR = 2.32, 95% CI = 1.78–3.02, *p* < 0.001). However, as no direct statistical contrast was performed between dual users and cigarette-only smokers, a definitive conclusion regarding the difference in risk levels between these two smoking groups cannot be drawn (Table 4).

**Table 4:** Impact of smoking type on metabolic syndrome.

Variables	Smoking type	OR	95% CI	*p*-value
Metabolic syndrome	Dual user	1.50	1.02–2.19	0.038
	Cigarette-only smoker	1.41	1.10–1.81	0.008
	Non-smoker	(reference)		
Abdominal obesity	Dual user	1.09	0.72–1.65	0.673
	Cigarette-only smoker	0.92	0.73–1.16	0.475
	Non-smoker	(reference)		
Hypertension	Dual user	0.86	0.54–1.37	0.518
	Cigarette-only smoker	0.86	0.67–1.09	0.210
	Non-smoker	(reference)		
High glucose	Dual user	1.13	0.77–1.67	0.535
	Cigarette-only smoker	1.12	0.89–1.41	0.321
	Non-smoker	(reference)		
Hypertriglyceridemia	Dual user	2.16	1.46–3.18	<0.001
	Cigarette-only smoker	1.91	1.54–2.36	<0.001
	Non-smoker	(reference)		
Low HDL cholesterol	Dual user	1.46	0.90–2.36	0.127
	Cigarette-only smoker	2.32	1.78–3.02	<0.001
	Non-smoker	(reference)		

CI, confidence interval; HDL, high-density lipoprotein; OR, odds ratio. Adjusted for sex, age, marital status, health insurance, education, household income, occupation, drinking, walking, stress, and depression.

## 4 Discussion

We examined the odds of MetS among cigarette smokers and dual users compared with that among non-smokers. Examination of the general characteristics of participants revealed that dual users were characterized by relatively high education levels and income, suggesting a higher socioeconomic status (SES) within this group. Consistent with previous findings on dual users in Korean population, Kim et al. (17) showed that current e-cigarette users had higher income and education levels than never-e-cigarette users. This pattern may be attributed to higher exposure to e-cigarette advertisements among high-SES individuals, which may facilitate their transition to dual use (23). In terms of occupation, a higher proportion of dual users were engaged in white-collar work than in blue-collar work. This pattern may be related to occupational characteristics; however, underlying reasons were not directly examined in this study.

Regarding health-related characteristics, the proportion of individuals with a smoking cessation plan was notably low among dual users. This may reflect that some dual users represent individuals in a transitional phase of quit attempts, potentially using e-cigarettes as a cessation aid (24). Furthermore, as dual users often perceive e-cigarette as less harmful than cigarettes (24, 25), they may perceive that they have effectively reduced their smoking risk. In terms of drinking behavior, both cigarette smokers and dual users exhibited a higher rate of frequent drinking than non-smokers. This suggests a clustering of health-risk behaviors, where smoking often co-occurs with high-risk drinking. While drinking was adjusted for in our model to isolate the independent association of smoking types with MetS, it should be noted that such lifestyle factors may also interact with smoking to influence metabolic health. Regarding mental health, dual users showed high levels of stress and depression compared with non-smokers, consistent with previous reports (17, 26). These patterns may be related to the demographic profile of dual users, who were often young and unmarried groups that may lack strong social support systems (27). While dual use is reportedly associated with increased levels of psychological distress (26), the temporal relationship between these factors cannot be confirmed in this cross-sectional study.”

Furthermore, we examined the odds of MetS among dual users and cigarette-only smokers relative to non-smokers, and found that both smoking groups showed significantly increased odds of MetS compared with non-smokers. Similar results have been reported previously. For example, Cai and Bidulescu reported an increased prevalence of MetS among dual users (13). Comprehensive meta-analyses and systematic reviews by Glantz et al. (14, 15) have also identified notable metabolic and cardiovascular indicators associated with dual use. Although some studies have reported varying results by sex (16, 17), and another reported no significant association between dual use and the risk of MetS (12), the overall body of evidence suggests that smoking patterns involving dual use may exhibit certain metabolic abnormalities relative to non-smoking. Given that half of the related previous studies involved Korean subjects, specific studies regarding the relationship between MetS and patterns of dual use by sex, race, and other factors are needed in the future, considering that population-specific determinants play a role in the epidemiology of MetS (2).

We also analyzed the five MetS components by smoking type. Considering abdominal obesity, there was no significant difference in the prevalence of abdominal obesity between the three groups, and the prevalence of abdominal obesity did not differ significantly between the groups. Reportedly, dual users may show a higher prevalence of abdominal obesity (12, 17, 28). In contrast, the absence of a correlation between dual users and abdominal obesity has also been reported (29, 30), consistent with the results of the current study. Considering the inconsistent results regarding the correlation between abdominal obesity and smoking type, additional studies are crucial to further verify the observed results.

Dual users showed relatively lower prevalence rates of hypertension and hyperglycemia than the other two smoking groups. These results are supported by previous studies that found no association between blood pressure and dual use (12, 13, 16, 17, 31) and by those that reported the absence of an association between blood glucose levels and dual use (12, 13, 17, 32). However, one study reported that only females were more likely to have hyperglycemia (16). Additional analyses are needed to comprehensively explore sex-based differences. The low prevalence of hypertension and hyperglycemia among dual users could be explained by demographic profile of the dual-user group, which predominantly consisted of younger adults, as shown in Table 1. Based on the results of this study and those reported previously, these findings suggest that there may be no clear association between dual use and hypertension and hyperglycemia.

Both dual users and cigarette-only smokers showed significantly higher odds of hypertriglyceridemia than non-smokers. While Cai and Bidulescu (13), identified significant associations with hypertriglyceridemia in dual users, and this finding is in line with the results of the current study. In addition, the results of Oh et al. (16) regarding female users were also observed in the current study. Conversely, while Kim et al. (12) showed no significant associations, Kim et al. (17) reported increased odds of hypertriglyceridemia among dual users relative to non-smokers, which is only partially consistent with our results.

In the current study, increased odds of low HDL cholesterol levels relative to non-smokers were observed among conventional cigarette users, whereas this association did not reach statistical significance in the dual user group. Although the results of previous studies by Kim et al. (12) and Oh et al. (16) were similar to those of the current study, other studies (13, 17) repotted that dual use was also associated with increased odds of low HDL levels compared to non-smokers, indicating that the results were inconsistent.

Considered in conjunction with the findings regarding the five MetS components, different smoking groups showed partially distinct patterns across individual MetS components; however, a clear explanation for these associations remains lacking. The underlying relationship between dual use and MetS is yet to be clearly elucidated, and extensive studies are needed to clarify this association. Additional studies are warranted to further investigate the status of abdominal obesity, hypertriglyceridemia, and low HDL levels, given the discrepancies in results between studies. Given the multiple comparisons performed across various MetS components, these analyses were considered exploratory.

We found that both dual users and cigarette-only smokers had increased odds of MetS compared with non-smokers. Recent and ongoing studies have also reported associations between smoking behaviors including dual use and cardiovascular disease (33–35). Nevertheless, many dual users may still have misconceptions regarding the safety of e-cigarettes (24, 25), and a substantially high proportion of individuals continue to use both e-cigarettes and conventional cigarettes (10–12). However, current policies for e-cigarettes are often less regulated than those for conventional cigarettes (8). Considering the results of the current study, global public health initiatives are crucial to improve awareness regarding the potential metabolic risks associated with smoking behaviors, including dual use.

This study has several limitations that warrant cautious interpretation. First, as a cross-sectional study, we could not determine the causal relationship between smoking types and MetS. Specifically, the possibility of reverse causality cannot be ruled out, for instance, individuals may have modified their smoking behaviors-such as transitioning to dual use-following medical advice or to reduce perceived health risks. Second, exclusive e-cigarette users were not analyzed as a separate group due to their insufficient sample size in the 2023 KNHANES dataset, which was inadequate for ensuring statistical power in an independent analysis. Consequently, the findings primarily reflect the characteristics of dual users rather than e-cigarette use in isolation, which may lead to potential exposure misclassification. Third, regarding our statistical approach, the inclusion of various health-related characteristics—such as alcohol consumption, physical activity, and mental health—as covariates might have introduced overadjustment bias, as some of these variables may lie on the causal pathway between smoking and metabolic syndrome. Fourth, the small dual-user sample (*n* = 161) may limit statistical power and risk model overfitting, despite adherence to EPV guidelines and diagnostics. Consequently, borderline significant associations warrant caution. Furthermore, because no formal correction for multiple testing was applied to individual MetS components, these findings should be interpreted as exploratory and hypothesis-generating. Fifth, the ‘non-smoker’ reference group included former smokers who may retain residual metabolic risks, potentially confounding the baseline risk assessment of the reference population. Finally, this study defined MetS according to the NCEP ATP-III criteria modified for the Korean population; which should be considered when generalizing the results. Future longitudinal studies are required to validate these associations and establish clear temporal relationships.

## 5 Conclusion

In conclusion, both dual users and cigarette-only smokers showed higher odds of MetS compared with non-smokers. Among the five components of MetS, hypertriglyceridemia was significantly associated with both smoking groups, whereas low HDL cholesterol was associated only with cigarette-only smokers. These findings suggest that smoking behaviors, including dual use, may associated with metabolic health risks relative to non-smoking. Public health strategies should continue to support smoking cessation and improve awareness regarding the potential metabolic risks associated with smoking behaviors.

## Data Availability

Publicly available datasets were analyzed in this study. This data can be found at: Korea National Health and Nutrition Examination Survey (KNHANES), provided by the Korea Disease Control and Prevention Agency (KDCA). Available at: https://knhanes.kdca.go.kr.
